# A Novel Nitrogen Metabolism Pathway in Strain *Gordonia* sp. TD-46: Genomic and Enzymatic Evidence

**DOI:** 10.3390/biology15100799

**Published:** 2026-05-17

**Authors:** Peiyang Zheng, Hao Li, Xiaojie Yan, Da Ao, Wenlong Yue, Shuai Zhang, Guanghua Yang, Zhiqiang Cai

**Affiliations:** College of Pharmacy, Changzhou University, Changzhou 213164, China; s23090817005@smail.cczu.edu.cn (P.Z.); s24030857031@smail.cczu.edu.cn (H.L.); s24020860008@smail.cczu.edu.cn (X.Y.); da_ao@cczu.edu.cn (D.A.); yuewenlong@cczu.edu.cn (W.Y.); zhangshuai@cczu.edu.cn (S.Z.)

**Keywords:** high-ammonia-nitrogen wastewater, whole-genome analysis, ammonia assimilation mechanism, assimilatory nitrate reduction

## Abstract

With the acceleration of industrialization, water pollution caused by ammonia-nitrogen compounds has become a severe environmental challenge. In recent years, significant breakthroughs in biological nitrogen removal technology have been achieved alongside the discovery of novel ammonia-nitrogen-degrading bacterial strains. This study delves into the metabolic pathways and molecular mechanisms of ammonia-nitrogen degradation by *Gordonia* sp. TD-46. A comprehensive understanding of the strain’s nitrogen metabolic pathways and the functions of key genes was achieved by optimizing its ammonia-nitrogen degradation conditions, analyzing its whole-genome sequence, and conducting heterologous expression of crucial genes. The results demonstrated that when ammonium chloride served as the nitrogen source, sodium acetate as the carbon source, with a C/N ratio of 25, pH of 7, and an inoculum size of 15%, the strain achieved an ammonia-nitrogen degradation rate exceeding 80% under these conditions. Whole-genome sequencing analysis identified genes involved in nitrogen metabolism, including *glnA*, *gdhA*, *narB*, *narGHI*, *nirBD*, and *nasBDE*. These genes indicate that the nitrogen metabolism pathway of strain TD 46 follows the assimilatory nitrate reduction pathway (NO_3_^−^ → NO_2_^−^ → NH_4_^+^) and the ammonia assimilation pathway (NH_4_^+^ → Gln → Glu). Thus, strain TD-46 is capable of efficient nitrogen assimilation.

## Simple Summary

Excessive ammonia nitrogen in water is a serious environmental problem that harms aquatic life and disrupts ecological balance. In this study, we discovered a bacterial strain, *Gordonia* sp. TD-46, which can remove up to 80% of ammonia nitrogen from water. By sequencing its genome, we identified a novel nitrogen metabolism pathway and confirmed it with key enzyme experiments. This work deepens our understanding of bacterial nitrogen metabolism and may lead to new, more effective biological solutions for cleaning ammonia-polluted water.

## 1. Introduction

As industrialization in China rapidly advances, the discharge of high-ammonia-nitrogen wastewater from sectors such as petrochemicals, pharmaceuticals, and aquaculture has become a critical challenge in water pollution control. Ammonia nitrogen, a key form of biologically active nitrogen, serves as a preferentially utilized nitrogen source for algae and photosynthetic bacteria. Its substantial input into water bodies—particularly in the presence of phosphorus—can significantly stimulate the abnormal proliferation of planktonic algae (e.g., cyanobacteria and diatoms), leading to algal blooms, depletion of dissolved oxygen, deterioration of water quality, and loss of biodiversity [1,2]. Furthermore, when untreated or inadequately treated high-ammonia-nitrogen wastewater is used for irrigation or leaks into soil, the nitrification of ammonia to nitrate can induce soil acidification and salinization, alter the structure of soil microbial communities, reduce soil fertility, and inhibit plant growth [3].

Currently, treatment technologies for high-ammonia-nitrogen wastewater are primarily categorized into physical, chemical, and biological methods, each with specific applicability, advantages, and limitations. Among these, biological methods offer advantages such as low operational costs and the absence of secondary pollution, making them the mainstream technology for modern wastewater treatment. However, for high-ammonia-nitrogen wastewater, traditional biological processes face challenges such as strong inhibitory effects and high carbon source demands. For instance, in the conventional nitrification–denitrification process, high concentrations of free ammonia exhibit significant inhibitory and toxic effects on nitrifying bacteria (*Nitrosomonas* and *Nitrobacter*) [4]. Specifically, *Nitrosomonas* is responsible for oxidizing ammonia (NH_3_) to nitrite (NO_2_^−^), while *Nitrobacter* is responsible for oxidizing nitrite to nitrate (NO_3_^−^). Both are nitrifying bacteria, and high free ammonia levels can inhibit both groups. This disrupts the normal progression of nitrification, leading to effluent with excessive ammonia-nitrogen and total nitrogen levels [5,6].

In recent years, significant breakthroughs have been achieved in novel biological nitrogen removal technologies. For example, partial nitrification–anammox (PN/A) involves oxidizing approximately 50% of NH_4_^+^ to NO_2_^−^ through partial nitrification, after which anammox bacteria directly convert the remaining NH_4_^+^ and the generated NO_2_^−^ into N_2_ [7,8,9]. Simultaneous nitrification and denitrification (SND) refers to the phenomenon where nitrification and denitrification occur concurrently within the same reactor. Its realization primarily relies on dissolved oxygen gradient control, creating a microenvironment with an aerobic outer layer and an anoxic inner layer within microbial flocs or biofilms [10,11]. The breakthroughs in these novel biological nitrogen removal processes are invariably dependent on the discovery of new nitrogen-removing microorganisms and novel nitrogen transformation pathways.

Heterotrophic nitrification–aerobic denitrification (HN-AD) bacteria are a class of specialized microorganisms capable of simultaneously completing nitrification and denitrification processes under aerobic conditions [12]. Diverging from traditional biological nitrogen removal theories, these microorganisms break through the limitation that nitrification and denitrification must occur under different oxygen conditions, exhibiting unique nitrogen metabolic characteristics. Research indicates that HN-AD bacteria mainly include genera such as *Pseudomonas*, *Alcaligenes*, and *Bacillus*, and their metabolic pathways involve complex enzyme systems and intricate regulatory mechanisms [13,14].

*Gordonia*, a genus of rare rod-shaped actinobacteria within the phylum Actinomycetota, has had its taxonomic position progressively clarified since its initial isolation from soil and clinical specimens by Tsukamura in 1971 [15]. Through 16S rRNA gene sequence analysis, Stackebrandt established it as the independent family Gordoniaceae, and to date, 41 valid species have been identified [16]. These species are widely distributed across complex habitats, including mangrove rhizospheres, petroleum-contaminated soil, wastewater, and human infection sites [17]. The cell wall of these strains contains mycolic acids (with carbon chain lengths of 40–66), and their genomic DNA exhibits a GC content of 63–69%. They are acid-fast positive and sensitive to lysozyme, displaying diverse colony morphologies (white, yellow, to red). These physiological characteristics confer a selective advantage for their isolation in high-salt or specific carbon source media [17,18,19,20,21].

Currently, the exploration of efficient ammonia-nitrogen-degrading bacterial strains remains insufficient, and the adaptability of reported strains to complex wastewater environments as well as their long term operational stability still await systematic verification. Moreover, most studies have focused on characterizing the nitrogen removal performance of isolates, while in-depth analyses of the molecular mechanisms underlying nitrogen metabolism and the functions of key enzyme systems are lacking. In this study, strain *Gordonia* sp. TD-46, isolated and preserved by the Laboratory of Applied Microbiology and Biotechnology at Changzhou University, was employed [22]. Preliminary experiments revealed that TD-46 did not produce NO_2_^−^ or NO_3_^−^ during ammonia-nitrogen degradation, leading us to hypothesize that TD-46 possesses a unique nitrogen metabolic pathway. To test this hypothesis, a one-factor-at-a-time experimental design was adopted to systematically investigate the effects of various culture conditions (including nitrogen source type, carbon source type, carbon-to-nitrogen ratio (C/N) ratio, pH, and inoculum size) on bacterial growth and ammonia-nitrogen metabolic performance. Through whole-genome analysis combined with functional gene identification, the nitrogen metabolic pathway of this strain was preliminarily characterized, and key genes involved in ammonia assimilation were heterologously expressed in *Escherichia coli* to verify their functions.

## 2. Materials and Methods

### 2.1. Strain, Reagents, and Culture Media

The microbial strain utilized in this study was isolated from a petrochemical wastewater treatment system in Maoming City, Guangdong Province. Following pre-enrichment and purification in the laboratory, the strain was identified as belonging to the genus *Gordonia* sp. and designated as strain TD-46. Strains TD-46, pET28a, *E. coli* DH5α, and BL21(DE3) are all preserved at the Laboratory of Applied Microbiology and Enzymology, Changzhou University [22].

Molecular biology reagents for strain identification were obtained from Shanghai Sangon Biotech Co., Ltd. (Shanghai, China), and polymerase chain reaction (PCR) primers were synthesized by Suzhou Genewiz Biotechnology Co., Ltd. (Suzhou, China). Molecular biology reagents for the gene heterologous expression experiments were purchased from Vazyme Biotech Co., Ltd. (Nanjing, China) and Takara Bio Inc. (Kusatsu, Japan).

Luria–Bertani (LB) medium (g/L): tryptone 10.0, yeast extract 5.0, NaCl 10.0, agar powder 2% (added for solid medium), pH 7.0, sterilized at 121 °C for 20 min.

Ammonia-nitrogen medium (g/L): NH_4_Cl 0.382, CH_3_COONa 2, MgSO_4_·7H_2_O 0.05, MnSO_4_·4H_2_O 0.01, FeSO_4_ 0.01, K_2_HPO_4_ 0.2, NaCl 0.12, pH 7.0–7.4, sterilized at 121 °C for 20 min [23,24,25].

### 2.2. Experimental Methods

#### 2.2.1. Method for the Determination of Nitrogen Content in Culture Media

To investigate the degradation of ammonia nitrogen in the fermentation medium by TD-46, experimental procedures were conducted in accordance with the national standard GB 7493-87 “Water quality—Determination of nitrite nitrogen—Spectrophotometric method” [26], the environmental protection standard HJ 536-2009 “Water quality—Determination of ammonia nitrogen—Salicylic acid spectrophotometric method” [27], and the environmental protection industry standard HJ/T 346-2007 “Water quality—Determination of nitrate nitrogen—Ultraviolet spectrophotometric method (Trial)” [28] to determine the concentrations of NO_2_^−^, NH_4_^+^, and NO_3_^−^ in the culture medium.

#### 2.2.2. Cultivation and Condition Optimization Experiments

In this study, the effects of carbon source type, nitrogen source type, C/N ratio, initial pH, and inoculum size on the growth and ammonia-nitrogen degradation efficiency of strain TD-46 were systematically investigated through single-factor experiments, and the optimal denitrification conditions were determined.

Strain TD-46 was inoculated into LB medium at an inoculum size of 1% (*v*/*v*) and cultured at 37 °C and 160 r/min for 24 h for activation. Subsequently, it was transferred to ammonia-nitrogen medium at an inoculum size of 10% (*v*/*v*) and cultured at 26 °C and 160 r/min.

To optimize the carbon source, glucose, sucrose, sodium acetate, sodium citrate, sodium succinate, and methanol were each used as the sole carbon source.

To optimize the nitrogen source, ammonium chloride, sodium nitrite, and potassium nitrate were each used as the sole nitrogen source.

To optimize the C/N ratio, experiments were conducted at C/N ratios of 2, 5, 7, 10, 15, 20, 25, 30, 35, 40, 45, 50, 60, 70, 80, 90, and 100.

To determine the optimal inoculum size for strain TD-46, optimization experiments were conducted with inoculum sizes set at 1%, 5%, 10%, 15%, 20%, and 25%.

To determine the optimal initial pH for strain TD-46, optimization experiments were conducted with pH set at 2, 3, 4, 5, 6, 7, 8, and 9.

Optical Density at 600 nm (OD_600_) was measured using a UV spectrophotometer (model EU-2200, Shanghai Angli Instruments Co., Ltd., Shanghai, China), and this value was positively correlated with the cell concentration.

#### 2.2.3. Identification and Whole-Genome Sequencing of Strain TD-46

Nucleic acid extraction of strain TD-46 was performed using the SanPrep Plasmid DNA Mini Extraction Kit (Sangon Biotech, Shanghai, China) according to the manufacturer’s instructions. The resulting qualified DNA products served as the initial template for subsequent PCR amplification.

The primers and PCR system for bacterial 16S rRNA amplification were used according to the literature [23].

The amplified PCR products were subjected to gel electrophoresis at 100 V for 30 min, and the resulting bands were observed to verify successful amplification. Using a sterile blade, gel slices containing the target DNA bands were excised from the agarose gel and purified with the SanPrep DNA Gel Extraction Kit (Sangon Biotech, Shanghai, China) strictly following the manufacturer’s protocol.

The purified products were then sent to Suzhou Genewiz Biotechnology Co., Ltd. (Suzhou, China). for sequencing. The obtained sequences were compared with the reference sequences available in the NCBI database, and a phylogenetic tree of the isolated strain was constructed using Mega 5.0 software.

Whole-genome sequencing of strain TD-46 was performed by Wuhan Bena Technology Service Co., Ltd. (Wuhan, China) using both Nanopore (long reads) and Illumina (short reads) sequencing platforms for in-depth analysis.

#### 2.2.4. Expression of *glnA*, *gdhA* in *Escherichia coli* BL21

The primers used for cloning the *glnA*, *gdhA* genes are listed in Table 1. The target genes were amplified by PCR and cloned into the pET28a vector via the In-Fusion method for expression. The recombinant plasmids were then transformed into *Escherichia coli* DH5α, and the cells were plated onto LB agar plates containing kanamycin (50 µg/mL) and incubated overnight at 37 °C. The following day, well-grown *E. coli* DH5α colonies were selected, and plasmids were purified using the SanPrep Plasmid Extraction Kit (Sangon Biotech, Shanghai, China) according to the manufacturer’s instructions. The isolated plasmids were verified by PCR using the universal T7 primers. Plasmids confirmed to be correct were subsequently transformed into *E. coli* BL21, and the cells were plated onto LB agar plates containing kanamycin (50 µg/mL) and incubated overnight at 37 °C. On the next day, well-grown *E. coli* BL21 colonies were selected and sent to Suzhou Genewiz Biotechnology Co., Ltd. for sequencing.

One milliliter of the recombinant *Escherichia coli* BL21 culture was inoculated into a 100 mL LB medium containing kanamycin (50 µg/mL) in a conical flask and incubated at 37 °C with shaking at 160 rpm for 2–3 h until the OD_600_ reached 0.6–0.8. IPTG was then added to a final concentration of 50 µM, and incubation was continued at 37 °C with shaking at 160 rpm for an additional 6 h. The culture was subsequently centrifuged at 6000 rpm for 10 min, and the cell pellet was resuspended and disrupted by ultrasonication for 30 min. The lysate was centrifuged at 12,000 rpm for 20 min at 4 °C to collect the supernatant, which was then analyzed by sodium dodecyl sulfate–polyacrylamide gel electrophoresis (SDS-PAGE).

#### 2.2.5. Purification of Glutamine Synthetase (GS) and Glutamate Dehydrogenase (GDH)

The crude enzyme solution obtained from induced expression was purified using a Ni-NTA 6FF column. The column was pre-equilibrated with a solution containing 20 mM Tris-HCl (pH 8.0). After pre-equilibration, the crude enzyme solution was loaded onto the Ni-NTA 6FF column and allowed to bind at 4 °C for 30 min. Following binding, non-target proteins were eluted using a solution containing 10 mM imidazole and 500 mM Tris-HCl (pH 8.0). Subsequently, target proteins were eluted with a gradient of imidazole concentrations ranging from 50 to 250 mM.

The purity and molecular weight of the proteins were analyzed by SDS-PAGE. Protein concentration was determined using the Bradford method with bovine serum albumin as a standard. The purification fold and recovery rate were calculated as follows:

Purification fold = Specific activity of purified enzyme/Specific activity of crude enzyme

Recovery rate (%) = (Total activity of purified enzyme/Total activity of crude enzyme) × 100%

#### 2.2.6. Assay of GS, GDH Enzyme Activities

GS activity was determined based on the principle that, in the presence of ATP and Mg^2+^, GS catalyzes the synthesis of glutamine from ammonium ions and glutamate. The resulting glutamine is further converted to γ-glutamyl hydroxamate, which forms a red complex with iron under acidic conditions. This complex exhibits a maximum absorbance at 540 nm, and the change in absorbance is used to quantify GS activity [29].

The assay was performed as follows: into a 1.5 mL centrifuge tube, 400 μL of reaction mixture (containing 12.24 g/L Tris, 19.66 g/L MgSO_4_·7H_2_O, 3.45 g/L sodium glutamate, 2.42 g/L L-cysteine hydrochloride, 0.76 g/L Ethylene Glycol Tetraacetic Acid (EGTA), and 5.56 g/L hydroxylamine hydrochloride, pH 7.4), 40 mmol/L ATP solution, and an appropriate volume of enzyme extract were added. The volume was adjusted to 750 μL with Phosphate-Buffered Saline (PBS) buffer (8 g/L NaCl, 0.2 g/L KCl, 1.42 g/L Na_2_HPO_4_, 0.27 g/L KH_2_PO_4_, pH 7.4). The mixture was vortexed thoroughly and incubated at 25 °C for 30 min. After incubation, 250 μL of color developing solution (23.176 g/L trichloroacetic acid, 101.02 g/L FeCl_3_·6H_2_O, and 50 mL/L concentrated hydrochloric acid) was added to terminate the reaction. The mixture was vortexed again, allowed to stand at room temperature for 10 min, and then centrifuged at 12,000 rpm for 5 min. The absorbance of the supernatant was measured at 540 nm. One unit (U) of GS activity was defined as the amount of enzyme that caused a 0.01 change in absorbance at 540 nm per minute per milligram of protein under the assay conditions.

GDH catalyzes the reductive amination of α-ketoglutarate using NH_4_^+^ and NADH, yielding glutamate and NAD^+^. Since NADH exhibits a maximum absorbance at 340 nm, the enzymatic reaction leads to a decrease in absorbance at this wavelength. GDH activity is therefore determined by monitoring the rate of decrease in absorbance at 340 nm [30].

For the assay, the following reagents were combined: 150 μL of 0.1 mol/L α-ketoglutarate, 150 μL of 1 mol/L (NH_4_)_2_SO_4_, 20 μL of 18 mmol/L ADP, 50 μL of 10 mmol/L NADH, and 50 μL of Tris-HCl buffer. The mixture was pre-incubated at 50 °C for 10 min, followed by the addition of 100 μL of enzyme extract. The decrease in absorbance at 340 nm was recorded within 1 min after the reaction start. One unit (U) of GDH activity was defined as the amount of enzyme required to oxidize 1 μmol of NADH per minute under the assay conditions, calculated using the molar extinction coefficient of NADH.

## 3. Results and Discussion

### 3.1. Strain Identification and Phylogenetic Tree Analysis

**Figure 1:**
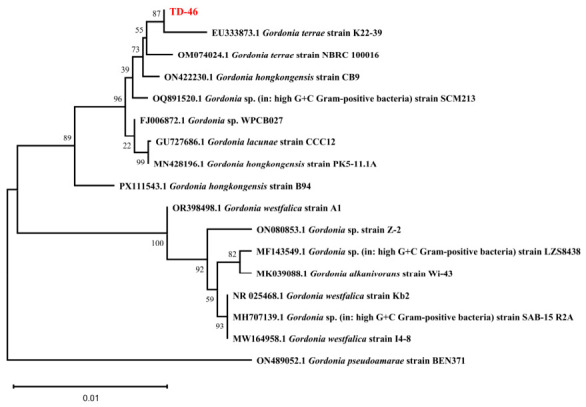
Phylogenetic tree of strain TD-46 (Strain TD-46 has been highlighted in red.).

Based on the colony morphology on solid medium, strain TD-46 forms large, orange-colored colonies that are non-flagellated, opaque, with a smooth and moist surface. Gram staining and observation under an optical microscope revealed that TD-46 is a Gram-positive bacterium with a rod-shaped morphology. Furthermore, based on the 16S rRNA gene sequencing results of strain TD-46, a phylogenetic tree was constructed using MEGA software (Version 11.0), as shown in Figure 1. The phylogenetic tree revealed that strain TD-46 exhibits the closest phylogenetic relationship with *Gordonia terrae* strain K22-39, with 100% sequence identity. By integrating the colony characteristics with the 16S rRNA sequence analysis, the taxonomic status of strain TD-46 was confirmed, identifying it as a member of the genus *Gordonia*.

### 3.2. Factors Influencing the Growth and Ammonia-Nitrogen Degradation of Strain TD-46

#### 3.2.1. Effect of Carbon Source Type on Strain TD-46

**Figure 2:**
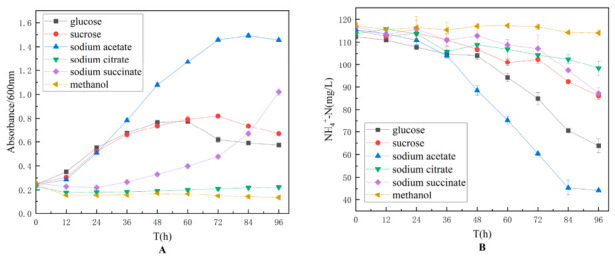
Effect of carbon source types on the growth (**A**) and nitrogen assimilation performance (**B**) of strain TD-46.

To optimize the carbon source, glucose, sucrose, sodium acetate, sodium citrate, sodium succinate, and methanol were each used as the sole carbon source. The experimental results are shown in Figure 2. Among the six carbon sources tested, sodium acetate was found to significantly enhance the ammonia-nitrogen degradation capacity of strain TD-46. Based on a comprehensive analysis of the strain’s growth characteristics, ammonia-nitrogen degradation efficiency, and metabolite accumulation patterns, sodium acetate was ultimately determined to be the optimal carbon source for strain TD-46.

It is noteworthy that strain TD-46 was unable to grow in the experimental groups using methanol or sodium citrate as the sole carbon source, presumably due to the absence of key metabolic enzyme systems, such as citrate lyase and methanol dehydrogenase, which are required for the effective breakdown and utilization of these carbon sources. As reported by Bekal et al., citrate lyase catalyzes the cleavage of citrate into oxaloacetate and acetate, representing the first and essential step in bacterial citrate metabolism [31]. Additionally, methanol dehydrogenase (MDH) catalyzes the oxidation of methanol to formaldehyde, a key rate-limiting step in methanol utilization by methylotrophic bacteria [32,33]. Strains lacking these functional enzymes, such as the *YlACL2*-disrupted mutant of *Yarrowia lipolytica*, have been shown to be incapable of utilizing citrate as a single carbon source [34]. Similarly, the loss of a functional methanol oxidation system results in the inability to grow on methanol as the sole carbon source [35].

#### 3.2.2. Effect of Nitrogen Source Type on Strain TD-46

**Figure 3:**
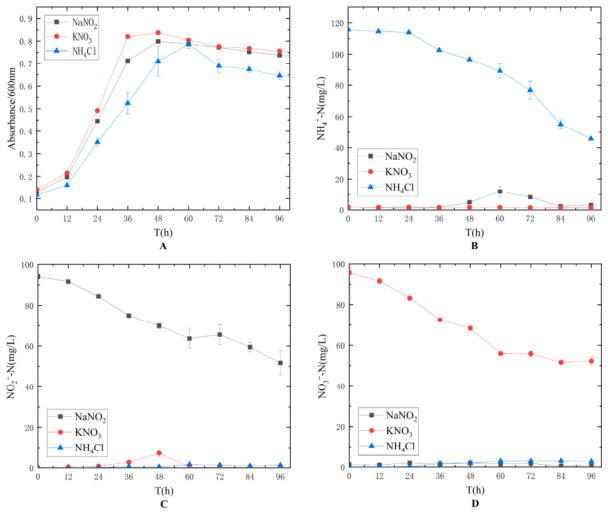
Effect of nitrogen source types on the growth (**A**) and nitrogen assimilation performance (**B**–**D**) of strain TD-46.

Experiments were conducted using ammonia nitrogen, nitrite nitrogen, and nitrate nitrogen as the sole nitrogen source, respectively, and the results are shown in Figure 3. It can be observed that strain TD-46 was able to utilize ammonia nitrogen, nitrite nitrogen, and nitrate nitrogen for growth. When ammonia nitrogen was used as the sole nitrogen source, no generation of nitrite nitrogen or nitrate nitrogen was detected. When nitrite nitrogen was the sole nitrogen source, ammonia nitrogen was generated. When nitrate nitrogen was the sole nitrogen source, nitrite nitrogen was generated. Based on these results, it can be preliminarily hypothesized that the nitrogen metabolic pathway of strain TD-46 does not follow the conventional sequence of NH_4_^+^ → NO_2_^−^ → NO_3_^−^ [13,36], and instead possesses a nitrate reduction pathway of NO_3_^−^ → NO_2_^−^ → NH_4_^+^ [37]. Further determination of the pathway requires subsequent genomic analysis.

#### 3.2.3. Effect of C/N Ratio on Strain TD-46

**Figure 4:**
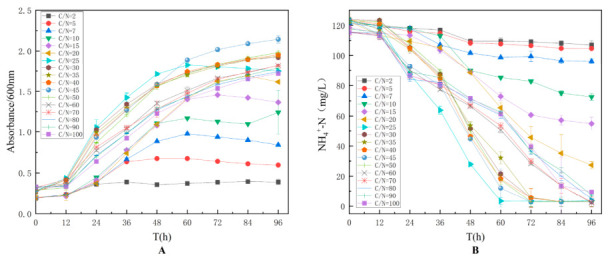
Effect of C/N ratio on the growth (**A**) and nitrogen assimilation performance (**B**) of strain TD-46.

To optimize the C/N ratio, experiments were conducted at C/N ratios of 2, 5, 7, 10, 15, 20, 25, 30, 35, 40, 45, 50, 60, 70, 80, 90, and 100. The results, as shown in Figure 4, indicated that the optimal ammonia-nitrogen degradation efficiency was achieved at a C/N ratio of 25. When the C/N ratio was below 10, strain TD-46 exhibited poor growth, and the ammonia-nitrogen degradation rate after 48 h was less than 30%. Therefore, it can be concluded that a low C/N ratio limits the growth of strain TD-46. However, at a C/N ratio of 100, the OD_600_ of strain TD-46 reached 1.4, indicating that the strain was still able to grow, with a final ammonia-nitrogen degradation rate of 80%. Based on these findings, the optimal C/N ratio for strain TD-46 was determined to be 25, and the strain demonstrated a high tolerance to elevated C/N ratios. This phenomenon may be associated with the salt tolerance of TD-46 [14,18]. The ability of TD-46 to maintain stable nitrogen assimilation activity under high C/N conditions could be attributed to the fact that an appropriately increased C/N ratio provides a more abundant carbon source for heterotrophic denitrifying bacteria. This promotes cell growth and energy metabolism (such as the TCA cycle and electron transport chain), thereby enhancing reductase activity and facilitating ammonia-nitrogen conversion [38].

#### 3.2.4. Effect of Inoculum Size on Strain TD-46

**Figure 5:**
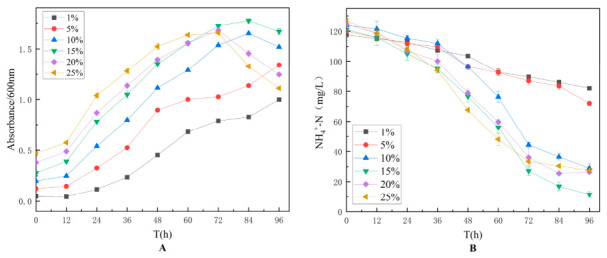
Effect of inoculum size on the growth (**A**) and nitrogen assimilation performance (**B**) of strain TD-46.

Inoculum size optimization was performed using inoculation volumes of 1%, 5%, 10%, 15%, 20%, and 25%, and the experimental results are presented in Figure 5. The results indicated that when the inoculum size was below 10%, the increase in OD_600_ was relatively slow, and ammonia-nitrogen degradation proceeded gradually. This suggests that a low inoculum size leads to slow growth of strain TD-46. At inoculum sizes above 20%, a sharp decline in OD_600_ was observed after 72 h, indicating that an excessively high inoculum size may result in rapid nutrient depletion and accumulation of metabolic wastes, thereby adversely affecting the growth of TD-46. An inoculum size of 15% was found to support robust growth of strain TD-46 and yielded high ammonia-nitrogen degradation efficiency.

#### 3.2.5. Effect of pH on Strain TD-46

**Figure 6:**
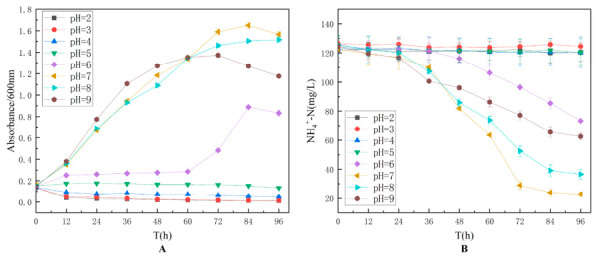
Effect of initial pH on the growth (**A**) and nitrogen assimilation performance (**B**) of strain TD-46.

pH optimization was conducted by adjusting the initial pH of the culture medium to 2, 3, 4, 5, 6, 7, 8, and 9, and the experimental results are shown in Figure 6. The results demonstrated that strain TD-46 was unable to grow under acidic conditions and exhibited optimal growth only in neutral to slightly alkaline environments. The strain showed the highest growth and ammonia-nitrogen degradation efficiency at pH 7. The superior performance of TD-46 under alkaline conditions may be attributed to its cellular membrane proton pump regulatory system. Previous studies have suggested that certain alkali-tolerant microorganisms maintain intracellular pH homeostasis through Na^+^/H^+^ antiporters [39].

### 3.3. Whole-Genome Sequencing and Analysis of Strain TD-46

#### 3.3.1. Genome Overview

In this study, sequencing was performed using the third-generation Nanopore sequencing platform and the second-generation Illumina sequencing platform to obtain a complete genome assembly of the bacterium, with a sequencing depth of ≥100×. After filtering out adapters, short fragments, and low-quality data, a total of 1,047,977,185 bp of clean data from the third-generation Nanopore sequencing was used for genome assembly, while the second-generation Illumina sequencing yielded 1,709,127,328 bp. The final assembled complete genome size was 5.499 Mb.

**Figure 7:**
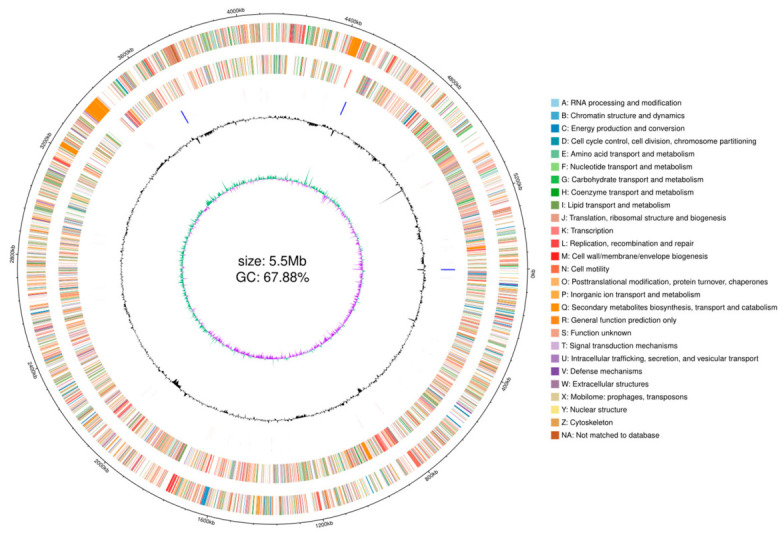
Circos plot of the genome of strain TD-46.

The circlize package in R (Version 0.4.18) was employed for the visualization and analysis of whole-genome sequencing data. This software effectively integrates multiple types of genomic feature data, including but not limited to sequencing depth distribution, GC content variation trends, and functional element annotations, enabling the simultaneous visualization of multidimensional data through a circular layout. The genome circle plot constructed using circlize intuitively visualizes the spatial relationships among different genomic elements, facilitating a systematic exploration of potential associations between genomic structure and functional characteristics. Figure 7 presents the circular map of the assembled genome, showing that strain TD-46 has a genome size of 5,498,545 bp with a GC content of 67.88%.

#### 3.3.2. Genome Functional Annotation

**Figure 8:**
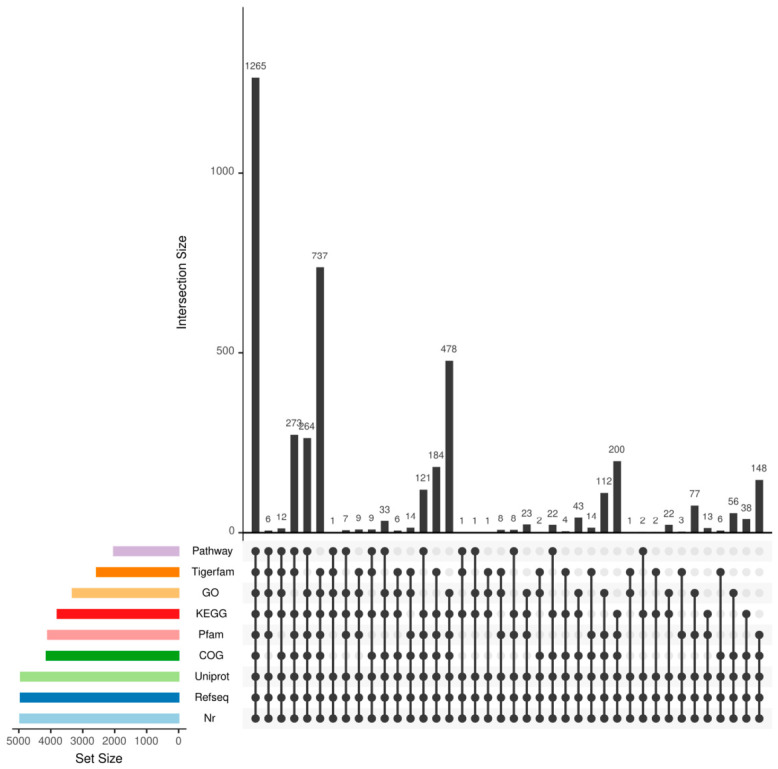
Statistical chart of shared and unique annotations of coding genes in common databases.

To comprehensively elucidate the functions of the genes, the predicted gene sequences were aligned against multiple functional databases using the BLAST+ (Version 2.17.0) algorithm to obtain functional annotation information. Additionally, complementary annotation analyses were performed with HMMER (Version 3.3.2) software based on the Pfam and TIGERFAM databases. The final functional annotation results are detailed in Figure 8 and Table 2.

##### Structural and Functional Classification Based on the Pfam Database

**Figure 9:**
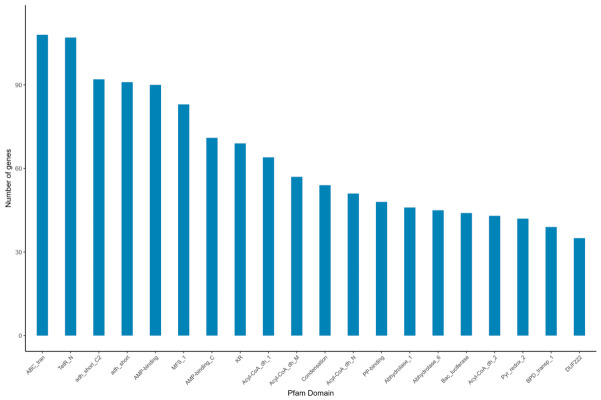
Statistical chart of PFAM domains.

Based on the annotations derived from the Pfam database, the genes corresponding to each domain were statistically summarized. The top 20 most frequently annotated domains were visualized in a bar chart, as shown in Figure 9.

The Pfam database is a collection of protein families, each represented by multiple sequence alignments and hidden Markov models (HMMs). In Figure 9, the horizontal axis represents the names of the protein families, and the vertical axis indicates the number of genes aligned to each respective protein family.

As shown in Figure 9 and Table 3, the gene sequences exhibited relatively high coverage of functional proteins, primarily including transporters, short-chain dehydrogenases, and membrane transport proteins. Notably, the Gln-synt_C domain of gene ctg_00591 is closely associated with the ammonium assimilation process, and the glutamine synthetase encoded by this gene plays a crucial role in facilitating ammonium assimilation in strain TD-46.

##### KEGG Pathway Classification

**Figure 10:**
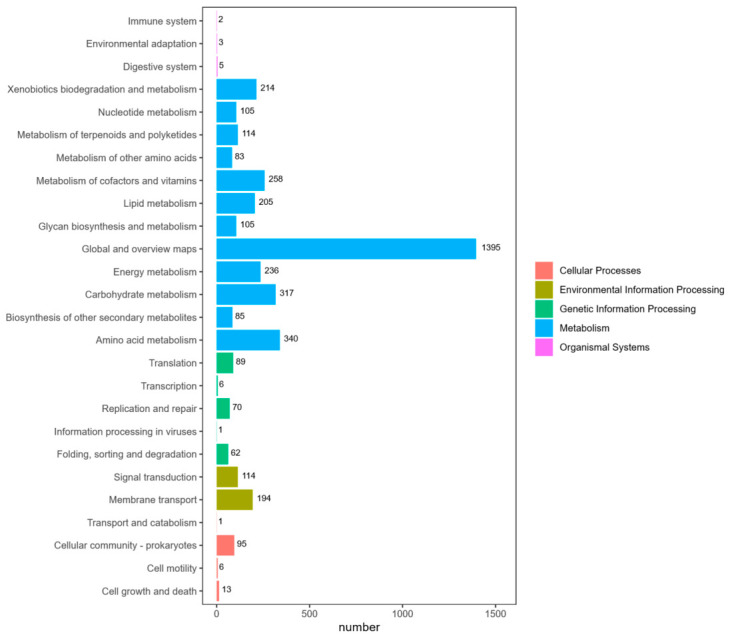
Classification chart of KEGG pathway results.

Functional annotation of the TD-46 genome based on the KEGG database was performed, and the distribution of genes involved in metabolic pathways at the secondary classification level is shown in Figure 10.

Among the metabolism-related genes, those assigned to global and overview maps were the most abundant (1395 genes), followed by genes involved in amino acid metabolism (340 genes), carbohydrate metabolism (317 genes), and metabolism of cofactors and vitamins (258 genes). Additionally, 214 genes were associated with xenobiotics biodegradation and metabolism, 194 genes encoded membrane transport proteins, and 95 genes were related to cellular community in prokaryotes.

The KEGG functional annotation of the TD-46 genome revealed that the number of metabolism-related genes significantly exceeded those in other functional categories, indicating that the core functions of strain TD-46 are predominantly focused on metabolic processes. The presence of a large number of genes involved in various metabolic activities within its genome may be a key factor contributing to the strain’s ability to adapt to complex environments and efficiently utilize resources [40]. Genes associated with environmental information processing and cellular processes were the next most abundant categories, suggesting that this strain is not only metabolically active but also possesses strong environmental adaptability and intercellular cooperation capabilities, enabling it to effectively sense and respond to external environmental changes and maintain its survival and growth through cellular processes [41]. Within the detailed distribution of metabolism-related genes, those involved in global and overview pathways were the most numerous, indicating that this strain possesses an extensive metabolic network capable of integrating multiple metabolic pathways to achieve efficient material and energy conversion. Furthermore, the presence of a substantial number of genes associated with xenobiotics biodegradation in TD-46 suggests that this strain has significant potential for pollutant degradation [17,19,20,21].

Partial database analysis results are provided in the Supplementary Materials (Table S1; Figures S1–S3).

#### 3.3.3. Identification of Functional Genes Involved in Nitrogen Metabolism

By integrating the KEGG annotation results from the whole-genome sequencing data of strain TD-46, the key functional genes involved in the nitrogen metabolic pathway were systematically identified. Table 4 presents the identification data of functional genes in strain TD-46, and these findings lay the foundation for in-depth analysis of the molecular mechanisms underlying the nitrogen metabolic pathway.

### 3.4. Nitrogen Metabolic Pathway of Strain TD-46

Based on the results of the effects of nitrogen sources on strain TD-46 and the identification of nitrogen metabolism-related functional genes, the nitrogen metabolic pathway of TD-46 was elucidated to follow the sequence: NO_3_^−^ → NO_2_^−^ → NH_4_^+^ → Gln → Glu [13]. This indicates that strain TD-46 possesses a complete nitrogen metabolism pathway.

**Figure 11:**
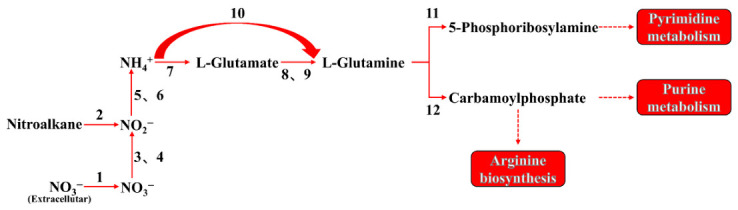
Nitrogen metabolism pathway of strain TD-46 (1: *nrt*, 2: *ncd*, 3: *narB*, 4: *narGHI*, 5: *nirBD*, 6: *nasBDE*, 7: *glnA*, 8: *glsA*, 9: *gltBD*, 10: *gdhA*, 11: *purF*, 12: *carA*).

Specifically, *nrtA* encodes a key transporter protein in the assimilatory nitrate reduction pathway. The resulting substrate-binding protein specifically captures extracellular nitrate and nitrite, transferring the substrate to other components of the ABC transporter for active transmembrane transport. [42]. The reduction of NO_3_^−^ to NO_2_^−^ is catalyzed by the enzymes encoded by *narB* and *narGHI* [14]. *narB* encodes an assimilatory nitrate reductase, whereas *narGHI* encodes a membrane-bound nitrate reductase; both enzymes facilitate the conversion of NO_3_^−^ to NO_2_^−^. The subsequent reduction of nitrite to NH_4_^+^ is mediated by the gene products of *nirBD* and *nasBDE* [43]. *nirBD* encodes a nitrite reductase, and *nasBDE* encodes an assimilatory nitrite reductase; both of which directly convert NO_2_^−^ to NH_4_^+^. Following ammonium generation, NH_4_^+^ is efficiently incorporated into glutamate via two distinct biochemical pathways orchestrated by the enzymes encoded by *glnA* and *gdhA*. Under low environmental ammonia concentrations, NH_4_^+^ is assimilated by glutamine synthetase (GS, encoded by *glnA*) to form glutamine (Gln), which is subsequently converted to glutamate (Glu) by glutamate synthase (GOGAT, encoded by *glsA* and *gltBD*). This ATP-dependent pathway exhibits a high affinity for ammonia, enabling effective capture and fixation even at micromolar levels. Conversely, when ammonia is abundant, glutamate dehydrogenase (GDH, encoded by *gdhA*) directly catalyzes the reversible conversion of NH_4_^+^ and α-ketoglutarate to glutamate without ATP consumption. However, due to its lower affinity for ammonia, GDH requires higher ambient ammonia concentrations for efficient activity [44]. This dual-system configuration allows strain TD-46 to adapt to environments with varying ammonia-nitrogen levels. The schematic diagram of this nitrogen metabolic pathway is shown in Figure 11.

Notably, strain TD-46 lacks both the *amo* genes, which encode ammonia monooxygenase for the conversion of NH_4_^+^ to NH_2_OH [23], and the *nxrAB* genes, encoding nitrite oxidoreductase for the conversion of NO_2_^−^ to NO_3_^−^. This genomic constitution indicates that TD-46 is incapable of performing complete nitrification. Consequently, the degradation of ammonia nitrogen in this strain does not proceed via the traditional nitrification–denitrification pathway but rather through a specialized ammonia assimilation mechanism.

In bacterial nitrogen metabolism, three major pathways are recognized. The first is the conventional autotrophic nitrification pathway, in which ammonia-oxidizing bacteria (e.g., *Nitrosomonas* sp.) oxidize NH_4_^+^ to NH_2_OH via *AmoA*, with subsequent conversion to NO_2_^−^ [45]; nitrite-oxidizing bacteria (e.g., *Nitrobacter* sp.) further oxidize NO_2_^−^ to NO_3_^−^ [46]; Complete ammonia oxidizers (e.g., *Nitrospira* sp.) can independently perform full nitrification (NH_4_^+^ → NO_2_^−^ → NO_3_^−^) [47]. All these processes generate nitrite and nitrate intermediates and release the greenhouse gases NO and N_2_O during ammonia oxidation. The second pathway is denitrification. Typical denitrifiers, such as *Paracoccus denitrificans*, reduce NO_3_^−^ sequentially to NO_2_^−^, NO, N_2_O, and N_2_ under anaerobic conditions [48]. Aerobic denitrifiers (e.g., *Pseudomonas stutzeri* XL-2) perform similar transformations under aerobic conditions but still emit some N_2_O and produce NO_2_^−^ and N_2_O as intermediates [49]. The third pathway is heterotrophic nitrification–aerobic denitrification. Bacteria such as *Alcaligenes faecalis*, *Paracoccus*, *Cupriavidus*, and *Pseudomonadaceae* oxidize NH_4_^+^ to NH_2_OH, NO_2_^−^, and NO_3_^−^ under aerobic conditions, which are then converted via denitrification to gaseous products (N_2_O/N_2_) [23,25,50,51]. This process also typically produces nitrite and nitrate intermediates. In summary, all three pathways generate nitrite, nitrate, or greenhouse gases to varying extents, thus posing a risk of secondary pollution.

The unique ammonia assimilation mechanism of strain TD-46 ensures that neither nitrate nor nitrite nitrogen is generated during the degradation process, thereby avoiding secondary pollution and highlighting its environmental friendliness.

### 3.5. Heterologous Expression of Key Nitrogen Metabolism Genes in Strain TD-46

#### 3.5.1. Expression of Target Genes

**Figure 12:**
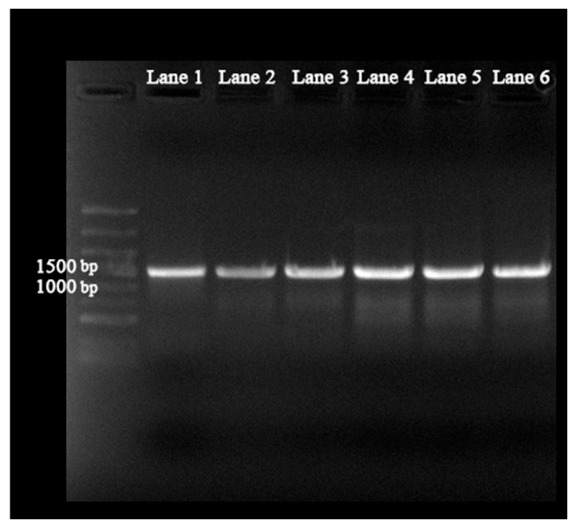
Agarose gel electrophoresis of PCR-amplified target genes (Lane 1–3: *glnA*; Lane 4–6: *gdhA*).

**Figure 13:**
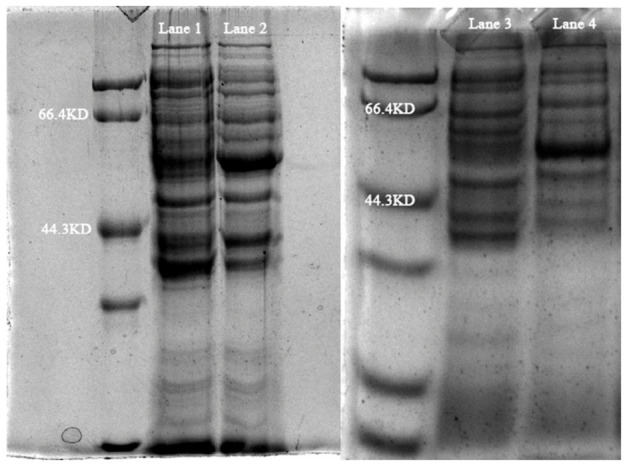
SDS-PAGE analysis of crude extract of GS and GDH (Lane 1, 3: Control group; Lane 2: GS; Lane 4: GDH).

As shown in Figure 12, total DNA of strain TD-46 was extracted and used as a template for PCR amplification of the *glnA* (1341) and *gdhA* (1362) genes. The amplified products were successfully ligated into the pET28a vector. The recombinant plasmids were transformed into competent *E. coli* BL21 cells, and the target proteins were successfully induced and expressed. The crude enzyme extract obtained after treatment was analyzed by SDS-PAGE, and the results are shown in Figure 13. The original gel electrophoresis figures and SDS-PAGE Figures are in Figure S4 of the Supplementary Material. The WB figures are in Supplementary File S1.

#### 3.5.2. Protein Purification and Enzyme Activity Assay

**Figure 14:**
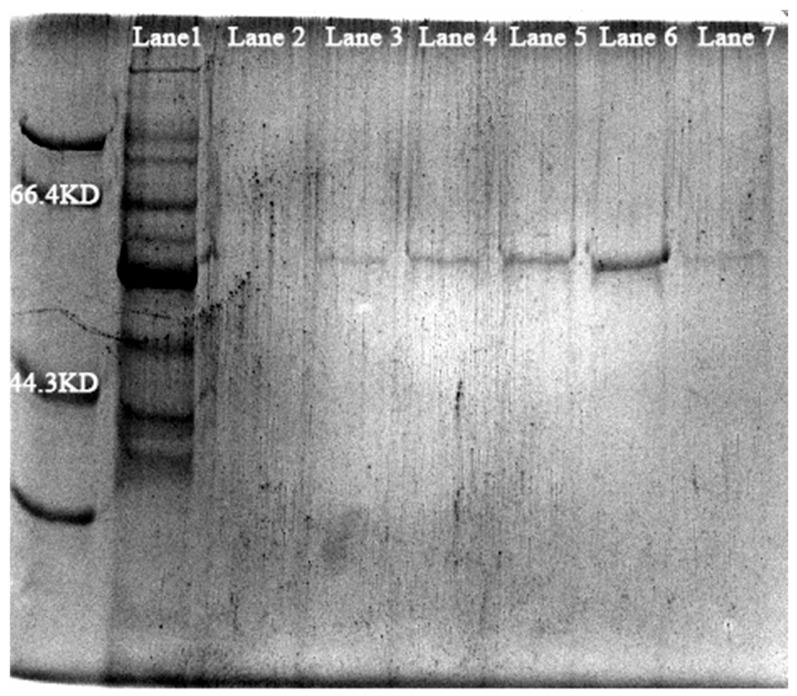
SDS-PAGE analysis of GS purification (Lane 1: Control group; Lane 2–7: 10, 20, 30, 40, 50, 60 mM imidazole eluent).

**Figure 15:**
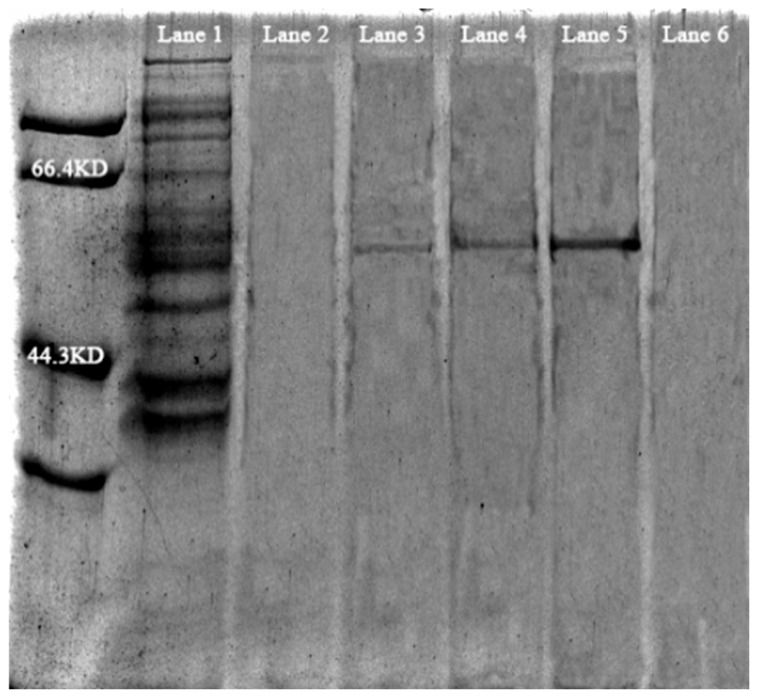
SDS-PAGE analysis of GS purification (Lane 1: Control group; Lane 2–6: 10, 20, 30, 40, 50 mM, imidazole eluent).

The results of protein purification are shown in Figure 14 and Figure 15. The original SDS-PAGE Figures are in Figure S4 of the Supplementary Material. SDS-PAGE analysis of the collected imidazole eluents at different concentrations revealed that the 50 mM imidazole eluent contained a distinct band corresponding to the target protein. The enzyme activity assay results of the purified protein are presented in Table 5.

The key genes *glnA* and *gdhA* involved in the nitrogen metabolism pathway of strain TD-46 were heterologously expressed in *Escherichia coli*. The expressed proteins GS and GDH, were subsequently purified and subjected to enzyme activity assays. The results directly confirmed that the ammonia-nitrogen metabolic pathway in strain TD-46 proceeds via NH_4_^+^ → Gln → Glu.

### 3.6. Limitations and Future Perspectives

(1) Salt tolerance mechanism: Genome annotation revealed that strain TD-46 possesses complete biosynthetic pathways for ectoine and glycine betaine, and salt tolerance tests confirmed its ammonia-nitrogen degradation ability at 30 g/L NaCl. However, multi omics approaches (transcriptomics, proteomics, metabolomics) were not employed to dissect the regulatory network. Future work should integrate these techniques to elucidate the adaptive mechanism under high salt conditions.
(2) Degradation of other pollutants: Strain TD-46 carries 214 genes related to xenobiotic biodegradation, consistent with the known pollutant degrading function of Gordonia. Nevertheless, this study did not perform degradation experiments for other pollutants (e.g., petroleum hydrocarbons, dyes). Future research should systematically evaluate its potential in the remediation of various pollutants.
(3) Incomplete enzymatic validation: The nitrogen metabolism of TD-46 relies on ammonia assimilation and assimilatory nitrate reduction. We successfully expressed the key ammonia assimilation genes (*glnA* and *gdhA*) heterologously, but some genes of the nitrate reduction pathway (encoding membrane proteins) could not be expressed. Future optimization of expression hosts or conditions is needed to validate the nitrate reduction pathway.
(4) Limitations of condition optimization and enzymatic characterization: The primary goal of this study was to elucidate the molecular mechanism of ammonia assimilation, not process optimization. Due to time constraints, only one-factor-at-a-time experiments (no response surface methodology) were used for condition screening. Moreover, for GS and GDH, only enzyme activities were measured; kinetic parameters and stability under different pH/temperature were not determined. Future studies should supplement these parameters to quantitatively evaluate the efficient ammonia assimilation capacity of TD-46.

## 4. Conclusions

In this study, the nitrogen assimilation performance of strain TD-46 was optimized. Through single-factor experiments, the optimal conditions for nitrogen assimilation by strain TD-46 were determined as follows: ammonium chloride as the nitrogen source, sodium acetate as the carbon source, a C/N ratio of 25, an initial pH of 7, and an inoculum size of 15%. Under these conditions, the ammonia-nitrogen degradation rate of strain TD-46 exceeded 80%.

Whole-genome sequencing analysis revealed that the genome size of strain TD-46 is 5.5 Mb, with a GC content of 67.88%, containing 5084 protein-coding genes. Genes related to ammonia assimilation (*glnA*, *gdhA*) and assimilatory nitrate reduction (*narB*, *narGHI, nirBD*, *nasBDE*) were identified, revealing the genetic basis for its unique and efficient nitrogen metabolism capability. Annotation also revealed that strain TD-46 possesses up to 214 genes associated with xenobiotics biodegradation, indicating its significant potential for pollutant degradation.

Through gene functional annotation and the heterologous expression of key genes (*glnA*, *gdhA*), it was elucidated that strain TD-46 achieves efficient nitrogen assimilation via the assimilatory nitrate reduction pathway (NO_3_^−^ → NO_2_^−^ → NH_4_^+^) and the ammonia assimilation pathway (NH_4_^+^ → Gln → Glu). Compared to the HN-AD pathway, this route does not produce nitrite or nitrate as secondary pollutants during ammonia-nitrogen degradation, demonstrating its environmental friendliness.

## Figures and Tables

**Table 1:** The primers used for cloning the *glnA*, *gdhA* genes.

Primers	The 5′→3′ Sequence
glnA-F	AATGGGTCGCGGATCCATGGATCGCCAAAAGGAATTCGT
glnA-R	GTGCGGCCGCAAGCTTCTACAGCGGCAGGTAGTTCTG
gdhA-F	AATGGGTCGCGGATCCGTGGTCGCCATCGTGGC
gdhA-R	GTGCGGCCGCAAGCTTTCAGATGAGACCCAGTGCAAGC

**Table 2:** Statistical summary of coding gene annotations.

Item	Count	Percentage
All	4977	100.00%
Annotation	4960	99.66%
KEGG	3790	76.15%
Pathway	2025	40.69%
Nr	4960	99.66%
Uniprot	4939	99.24%
GO	3317	66.65%
COG	4133	83.04%
Pfam	4090	82.18%
Refseq	4943	99.32%
Tigerfam	2561	51.46%

**Table 3:** PFAM domain annotation.

SeqID	Accession	HMMProfile	Description
ctg_00048	PF00005.32	ABC_trangg	ABC transporter
ctg_00063	PF00106.30	adh_short	short-chain dehydrogenase
ctg_00063	PF13561.11	adh_short_C2	Enoyl-(Acyl carrier protein) reductase
ctg_00591	PF12437.13	GSIII_N	Glutamine synthetase type III N terminal
ctg_00591	PF00120.29	Gln-synt_C	Glutamine synthetase, catalytic domain
ctg_00592	PF18318.6	Gln-synt_C-ter	Glutamine synthetase C-terminal domain
ctg_03819	PF03951.24	Gln-synt_N	Glutamine synthetase, beta-Grasp domain
ctg_00521	PF03460.22	NIR_SIR_ferr	Nitrite/Sulfite reductase ferredoxin-like half domain
ctg_00521	PF01077.27	NIR_SIR	Nitrite and sulfite reductase 4Fe-4S domain
ctg_03152	PF02665.19	Nitrate_red_gam	Nitrate reductase gamma subunit
ctg_03153	PF02613.20	Nitrate_red_del	Nitrate reductase delta subunit
ctg_03154	PF14711.11	Nitr_red_bet_C	Respiratory nitrate reductase beta C-terminal
ctg_04976	PF21075.2	GDH_ACT1	Glutamate dehydrogenase, ACT1 domain
ctg_04976	PF21073.2	GDH_HM1	Glutamate dehydrogenase, helical motif 1
ctg_04976	PF21076.2	GDH_ACT2	Glutamate dehydrogenase, ACT2 domain
ctg_04976	PF21077.2	GDH_ACT3	Glutamate dehydrogenase, ACT3 domain
ctg_04976	PF21078.2	GDH_HM3	Glutamate dehydrogenase, helical motif 3

**Table 4:** Identification of functional genes.

Functional Genes	Key Emzymes	Functions	Identification Results
*nrtA*	Nitrite transport system substrate-binding protein	Extracellular NO_3_^−^ Intracellular NO_3_^−^	+
*narB*	Ferredoxin–nitrate reductase	NO_3_^−^-NO_2_^−^	+
*narGHI*	nitrate reductase	NO_3_^−^-NO_2_^−^	+
*nirBD*	nitrite reductase (NADH)	NO_2_^−^-NH_4_^+^	+
*nasBDE*	nitrite reductase [NAD(P)H]	NO_2_^−^-NH_4_^+^	+
*glnA*	glutamine synthetase	Gln-Glu	+
*gltBD*	glutamate synthase (NADPH)	Gln-Glu	+
*glsA*	glutaminase	Gln-NH_4_^+^	+
*gdhA*	glutamate dehydrogenase (NADP^+^)	NH_4_^+^-Gln	+
*purF*	amidophosphoribosyltransferase	Glu-5-PRA	+
*carAB*	Carbamoyl-phosphate synthase	Glu-CP	+
*ncd2*	Nitronate monooxygenase	Nitroalkene-NO_2_^−^	+

**Table 5:** Enzyme activity assay.

Sample	Total Activity (U)	Protein Content (mg)	Specific Activity (U/mg)	Purification Fold	Recovery Yield (%)
Crude extract (GS)	4.70	1.37	3.43	-	-
GS	3.19	0.21	15.19	4.43	67.9
Crude extract (GDH)	5.66	1.88	3.01	-	-
GDH	4.88	0.33	14.79	4.91	86.2

## Data Availability

Data will be made available on request.

## Supplementary Materials

The following supporting information can be downloaded at: https://www.mdpi.com/article/10.3390/biology15100799/s1, Table S1: Statistics of Gene Prediction Results; Figure S1: Classification Chart of GO Annotation Results; Figure S2: COG Functional Classification of Strain TD-46; Figure S3: Species Distribution Chart of Strain TD-46 Based on NR Database; Figure S4: The Original Gel Electrophoresis Figures and SDS-PAGE Figures. File S1: WB figures.

## Abbreviations

The following abbreviations are used in this manuscript:

C/N ratio	carbon-to-nitrogen ratio
PN/A	partial nitrification–anammox
SND	Simultaneous nitrification and denitrification
HN-AD	Heterotrophic nitrification–aerobic denitrification
PCR	polymerase chain reaction
LB	Luria–Bertani
OD_600_	Optical Density at 600 nm
IPTG	Isopropyl β-D-1-thiogalactopyranoside
SDS-PAGE	sodium dodecyl sulfate–polyacrylamide gel electrophoresis
GS	Glutamine synthetase
GDH	Glutamate dehydrogenase
EGTA	Ethylene Glycol Tetraacetic Acid
PBS	Phosphate-Buffered Saline
