# Supplementary material for: A Novel Nitrogen Metabolism Pathway in Strain Gordonia sp. TD-46: Genomic and Enzymatic Evidence
Source: Biology (Basel). 2026 May 17;15(10):799. doi: 10.3390/biology15100799 (PMC13203658; doi:10.3390/biology15100799)
Supplement: Supplementary file 1 [file biology-15-00799-s001.zip › Figure S1. Classification Chart of GO Annotation Results.pdf]

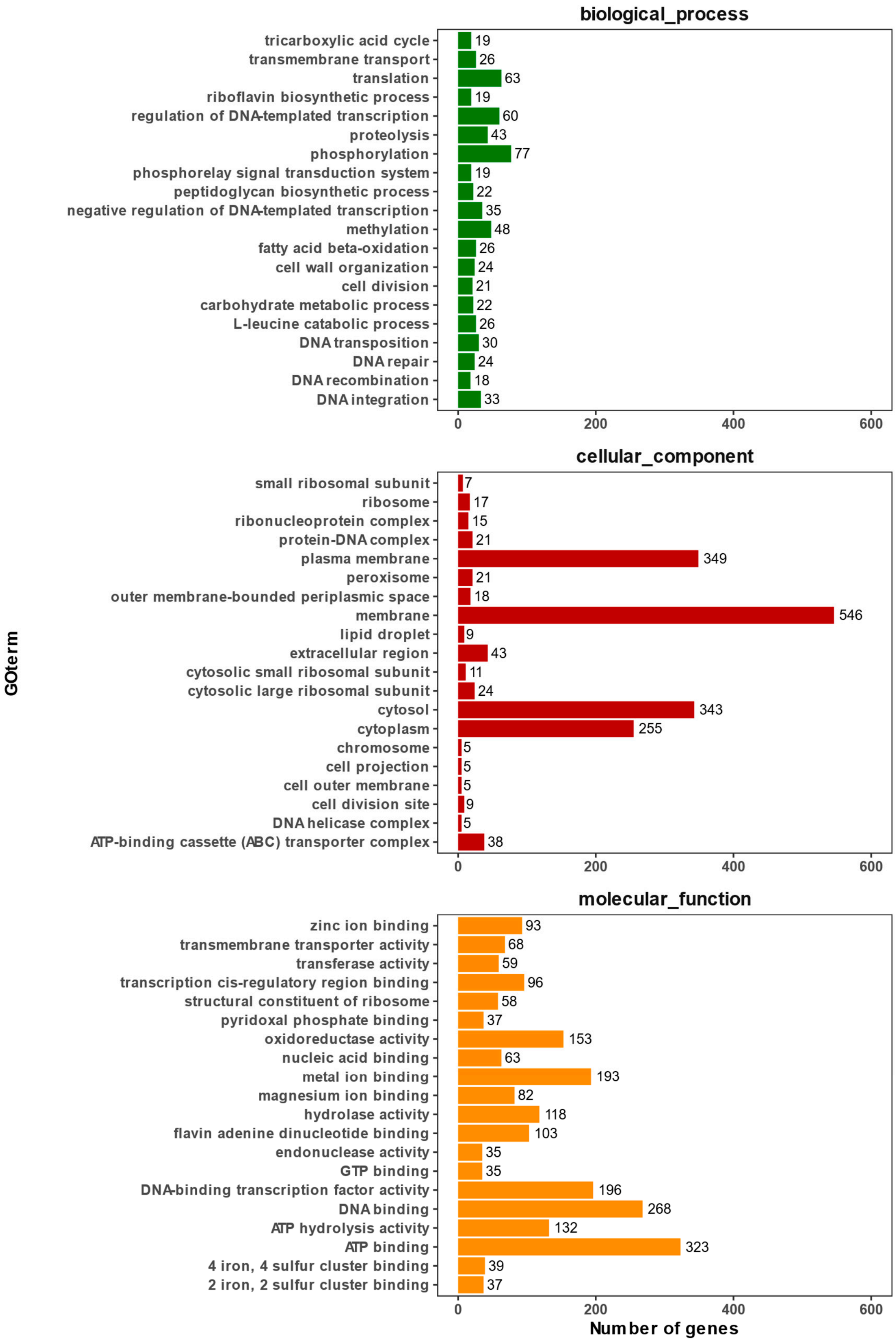

Figure S1. Classification Chart of GO Annotation Results

In this study, the GO annotation information of the genes was simplified to obtain GOslim classification results. A systematic analysis of gene function was conducted from three perspectives: cellular component, molecular function, and biological process.

After quantifying the number of genes assigned to each category, the top 20 most frequently annotated GOslim terms in each main category were selected for visualization(Figure 10). In this bar chart, the horizontal axis represents the GO classification terms, while the vertical axis corresponds to the distribution characteristics of genes at the GO secondary functional level under different backgrounds, thereby revealing the relative importance of different functional categories in specific biological contexts.

The analysis results showed that within the biological process category, phosphorylation was the most abundant, involving 77 genes. In terms of cellular components, genes associated with membrane parts accounted for the highest proportion, totaling 546 genes. Regarding molecular function, genes with ATP binding, DNA binding, and metal ion binding activities were the most prevalent, with counts of 323, 268, and 193 genes, respectively. These findings clearly indicate that strain TD-46 possesses significant metabolic activity and strong growth potential.
