# Supplementary material for: A Novel Nitrogen Metabolism Pathway in Strain Gordonia sp. TD-46: Genomic and Enzymatic Evidence
Source: Biology (Basel). 2026 May 17;15(10):799. doi: 10.3390/biology15100799 (PMC13203658; doi:10.3390/biology15100799)
Supplement: Supplementary file 1 [file biology-15-00799-s001.zip › Figure S2. COG Functional Classification of Strain TD-46.pdf]

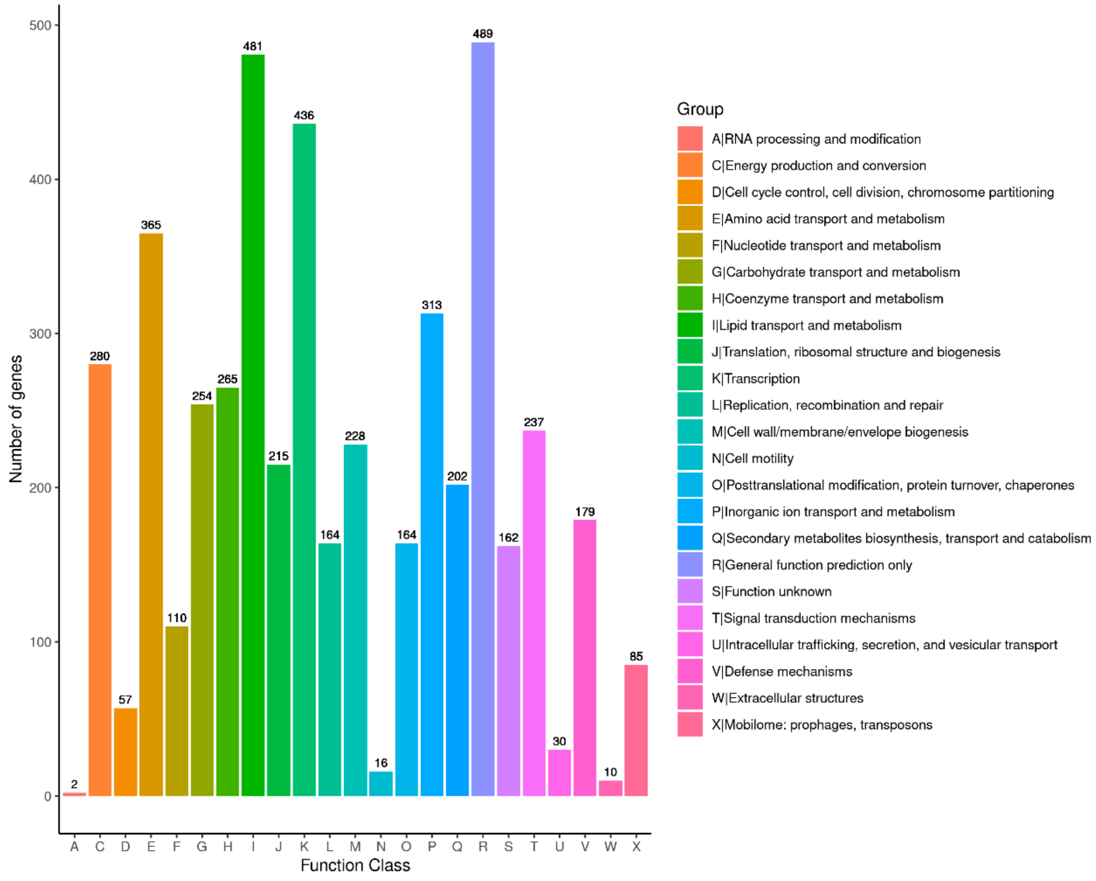

Figure S2. COG Functional Classification of Strain TD-46

Based on the classification criteria of the Clusters of Orthologous Groups (COG) database, all genes were categorized into 26 functional groups. As shown in the figure12, the differences in gene counts across various functional categories reflect the metabolic characteristics under specific physiological periods or environmental conditions, providing an important basis for the physiological function analysis of the research subject.

The gene functional annotation results indicate that these genes are primarily involved in the following biological processes: RNA processing and modification, energy production and conversion, and amino acid transport and metabolism. Notably, a large number of genes related to lipid transport and metabolism were identified, totaling 481. This phenomenon suggests that lipids play a central role in cell structure construction, energy acquisition, and environmental adaptation for strain TD-46. By modulating the saturation, chain length, or type of membrane lipids (such as synthesizing specialized phospholipids or ether lipids), TD-46 maintains membrane fluidity to cope with envi-ronmental stresses including temperature fluctuations, osmotic pressure, and salinity variations, further substantiating the strong salt

tolerance of this strain.

Furthermore, high gene abundance was observed across multiple functional categories, including general function prediction only, transcription, amino acid transport and metabolism, and inorganic ion transport and metabolism. These functional genes are primarily involved in essential physiological processes such as electron transport chains and cellular signal transduction.
