# Supplementary material for: A Novel Nitrogen Metabolism Pathway in Strain Gordonia sp. TD-46: Genomic and Enzymatic Evidence
Source: Biology (Basel). 2026 May 17;15(10):799. doi: 10.3390/biology15100799 (PMC13203658; doi:10.3390/biology15100799)
Supplement: Supplementary file 1 [file biology-15-00799-s001.zip › Figure S3 Species Distribution Chart of Strain TD-46 Based on NR Database.pdf]

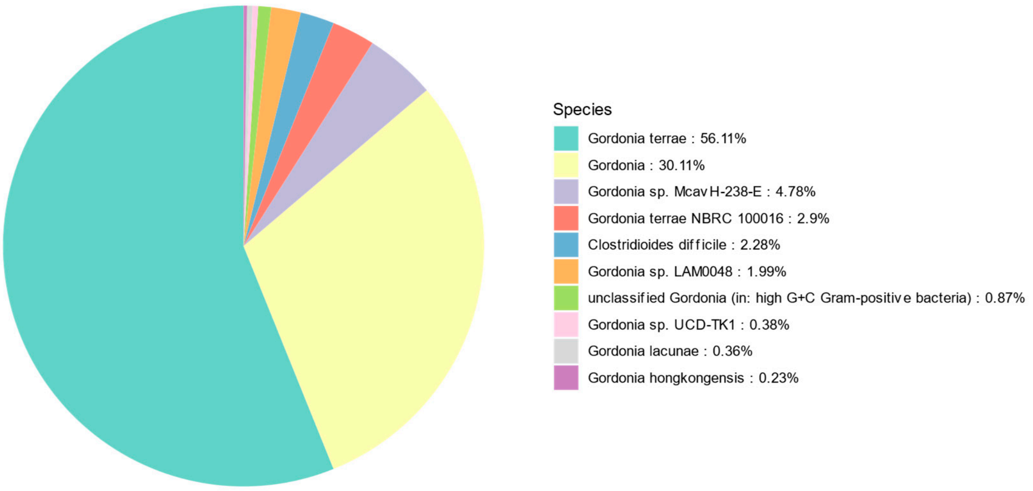

Figure S3. Species Distribution Chart of Strain TD-46 Based on NR Database

Functional annotation of the strain TD-46 genome based on the NR database was performed, and the results are shown in the figure13, with different colors representing different species. Protein sequence alignment revealed that 56.11% of the protein se-quences encoded by this strain were highly homologous to those of Gordonia terrae. This molecular-level evidence further supports the taxonomic classification of strain TD-46 within the genus Gordonia.
