# Supplementary material for: A Novel Nitrogen Metabolism Pathway in Strain Gordonia sp. TD-46: Genomic and Enzymatic Evidence
Source: Biology (Basel). 2026 May 17;15(10):799. doi: 10.3390/biology15100799 (PMC13203658; doi:10.3390/biology15100799)
Supplement: Supplementary file 1 [file biology-15-00799-s001.zip › Figure S4. the original Gel electrophoresis figures and SDS-PAGE figures .pdf]

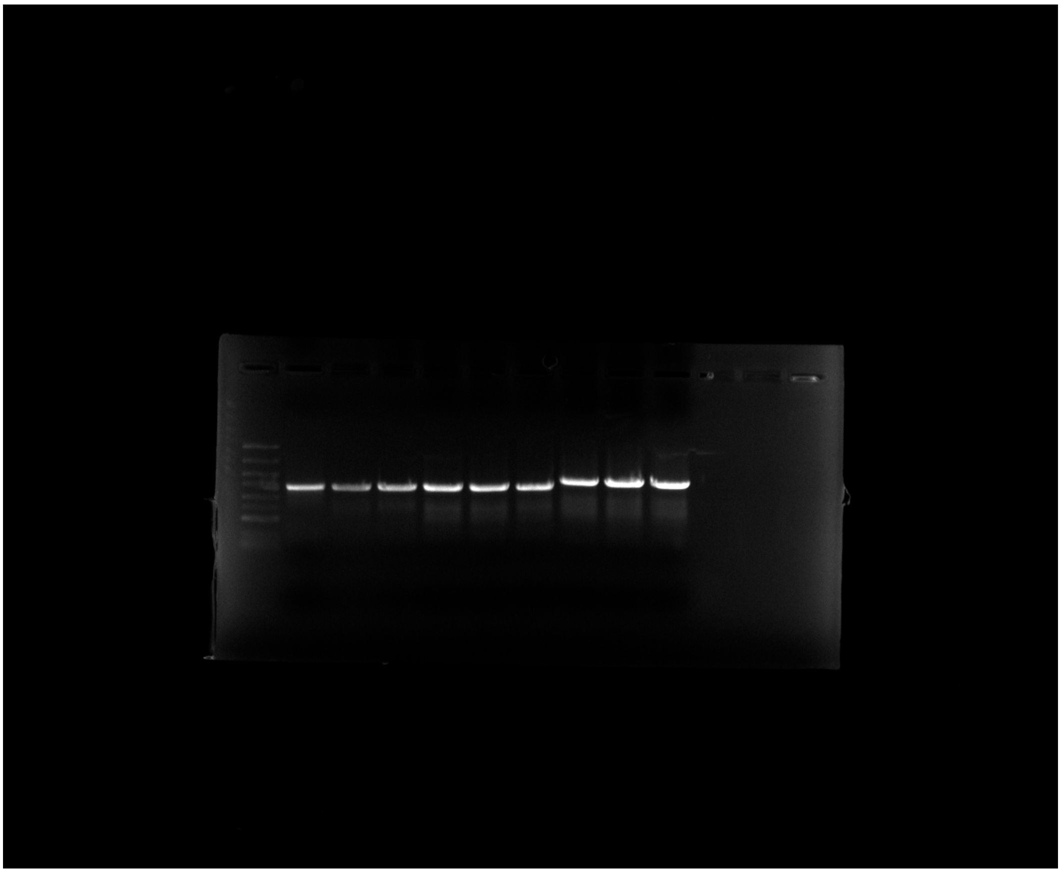

Figure S4-1. Agarose Gel Electrophoresis of PCR-Amplified Target Genes.

Lane 1 is the DNA marker, lanes 2 – 4 are the *glnA* gene, lanes 5 – 7 are the *gdhA* gene, and lanes 8 – 10 are the *narB* gene.

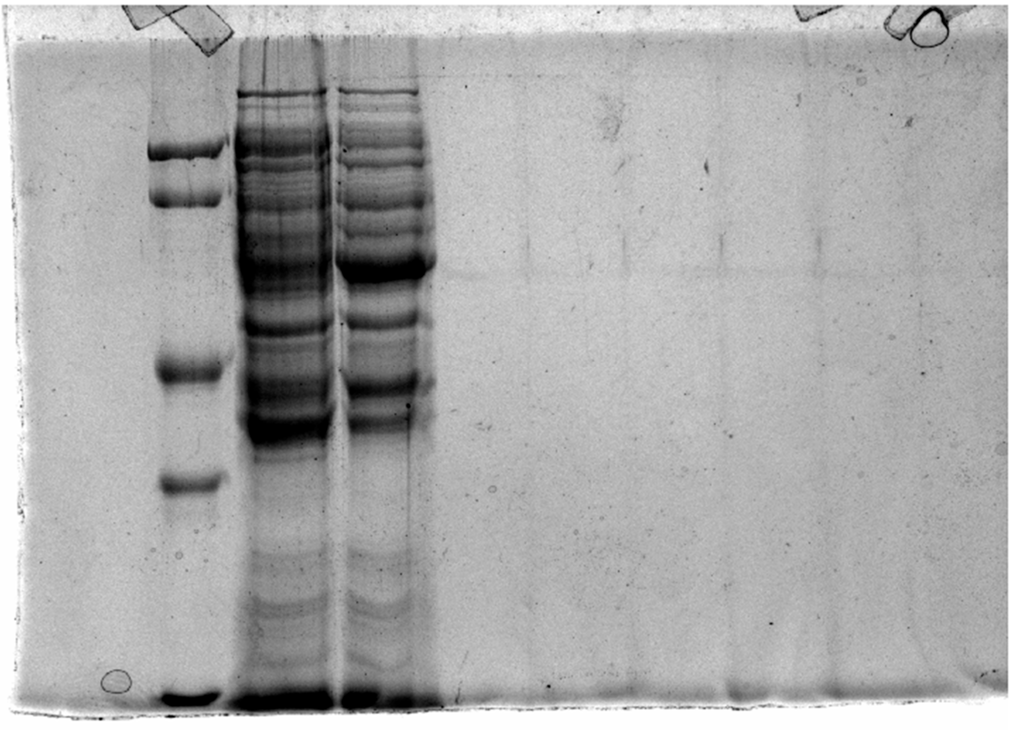

Figure S4-2. SDS-PAGE Analysis of Crude Extract of GS

Lane 1 is the protein marker, lane 2 is the control group, and lane 3 is the crude GS enzyme.

The control group is *Escherichia coli* that has not been transformed with the *glnA* gene.

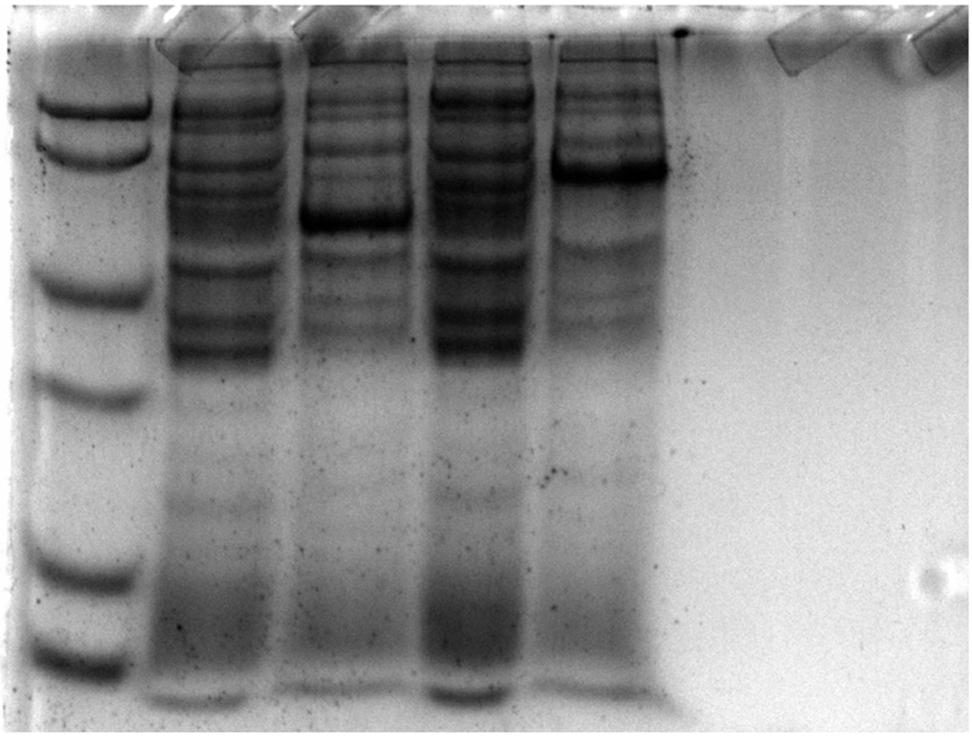

Figure S4-3. SDS-PAGE Analysis of Crude Extract of GDH

Lane 1 is the protein marker, lane 2 is the control group, lane 3 is the crude GDH enzyme, lane 4, 5 is another protein that is not relevant to this study.

The control group is *Escherichia coli* that has not been transformed with the *gdhA* gene.

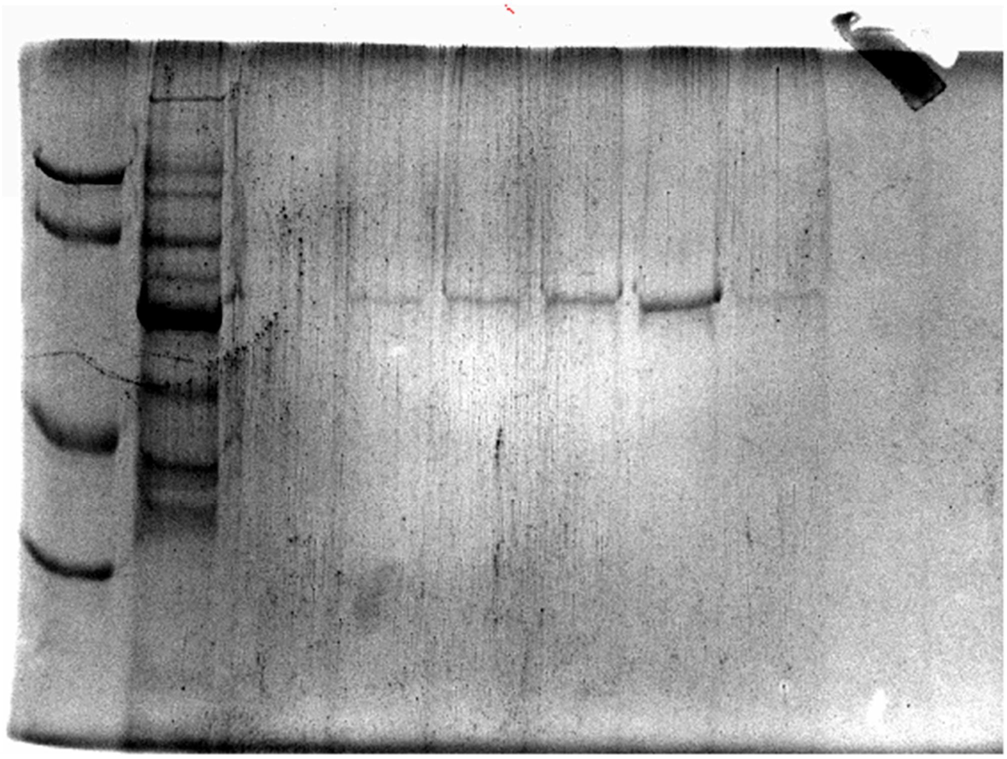

Figure S4-4. SDS-PAGE Analysis of GS Purification

Lane 1 is the protein marker, lane 2 is the control group, and lanes 3–8 are elution fractions with 10, 20, 30, 40, 50, and 60 mM imidazole

The control group is the crude GS enzyme

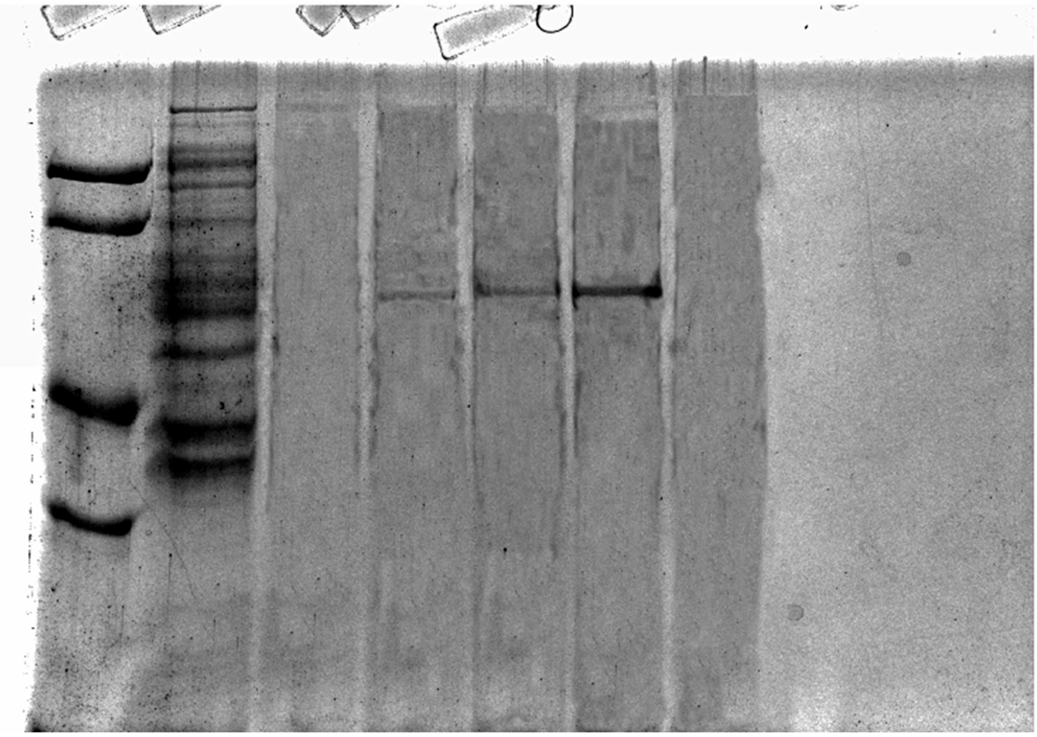

Figure S4-5. SDS-PAGE Analysis of GDH Purification

Lane 1 is the protein marker, lane 2 is the control group, and lanes 3–7 are elution fractions with 10, 20, 30, 40, and 50 mM imidazole

The control group is the crude GDH enzyme
