# Supplementary material for: A Novel Nitrogen Metabolism Pathway in Strain Gordonia sp. TD-46: Genomic and Enzymatic Evidence
Source: Biology (Basel). 2026 May 17;15(10):799. doi: 10.3390/biology15100799 (PMC13203658; doi:10.3390/biology15100799)
Supplement: Supplementary file 1 [file biology-15-00799-s001.zip › Table S1 Statistics of Gene Prediction Results.pdf]

Table S1. Statistics of Gene Prediction Results

Type	Number	Total_len	Average_len	Percentage of genome(%)
Gene	5,084	5,001,508	984	90.96
CDS	4,977	4,976,889	1,000	90.51
tRNA	53	4,208	79	0.08
23S rRNA	3	9,378	3,126	0.17
16S rRNA	3	4,563	1,521	0.08
5S rRNA	3	306	102	0.01
tmRNA	1	372	372	0.01
misc_rna	44	5,792	132	0.11

In this study, the assembled genome sequence was annotated using Prokka (version 1.14.6). This software integrates multiple bioinformatics tools for gene prediction and annotation: Prodigal was employed for the identification of protein-coding genes, Aragorn for tRNA prediction, RNAmmer for rRNA annotation, and Infernal for the detection of other non-coding RNAs. The results from each prediction module were systematically integrated to generate comprehensive genome annotation information. The detailed prediction data are presented in Table S1.
