# Supplementary figures and images for: A Novel Nitrogen Metabolism Pathway in Strain Gordonia sp. TD-46: Genomic and Enzymatic Evidence
Source: Biology (Basel). 2026 May 17;15(10):799. doi: 10.3390/biology15100799 (PMC13203658; doi:10.3390/biology15100799)

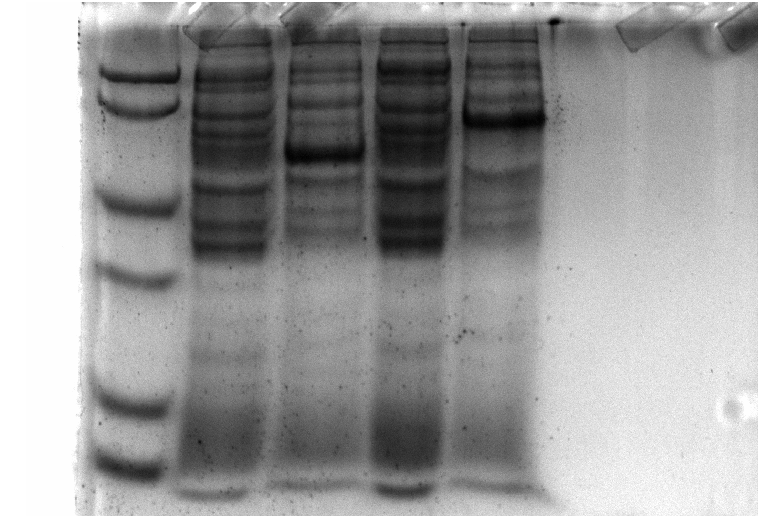

Supplement: Supplementary file 1 [file biology-15-00799-s001.zip › File S1. WB figures/crude GDH enzyme.tif]

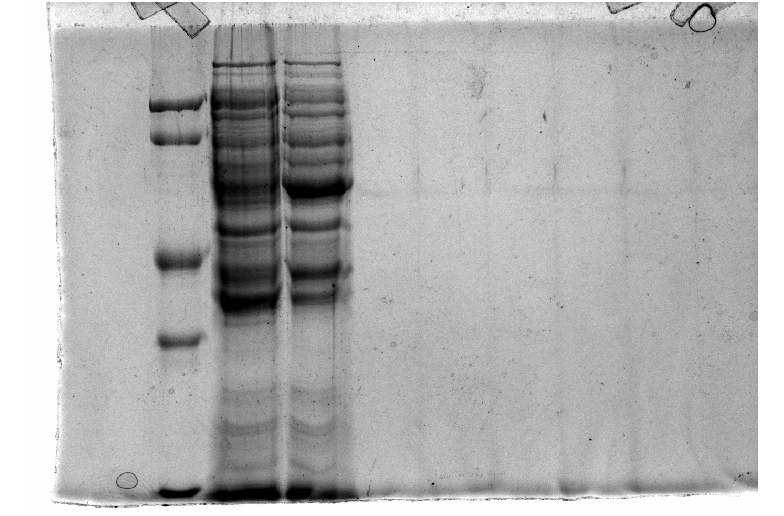

Supplement: Supplementary file 1 [file biology-15-00799-s001.zip › File S1. WB figures/crude GS enzyme.tif]

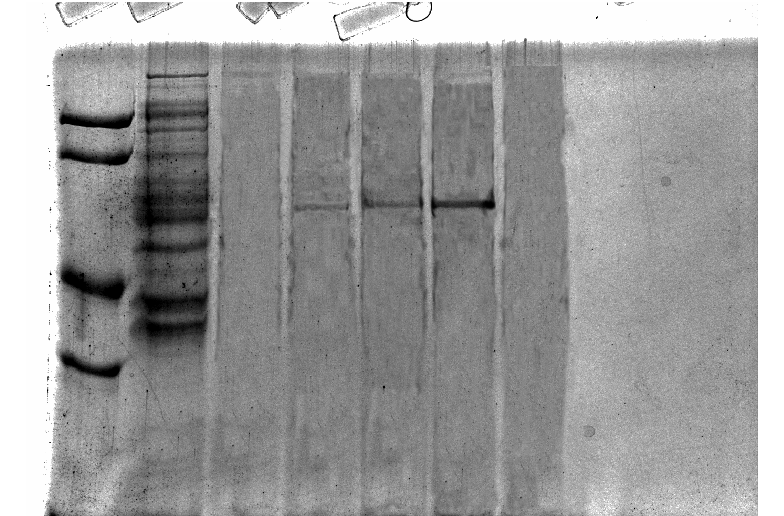

Supplement: Supplementary file 1 [file biology-15-00799-s001.zip › File S1. WB figures/GDH enzyme.tif]

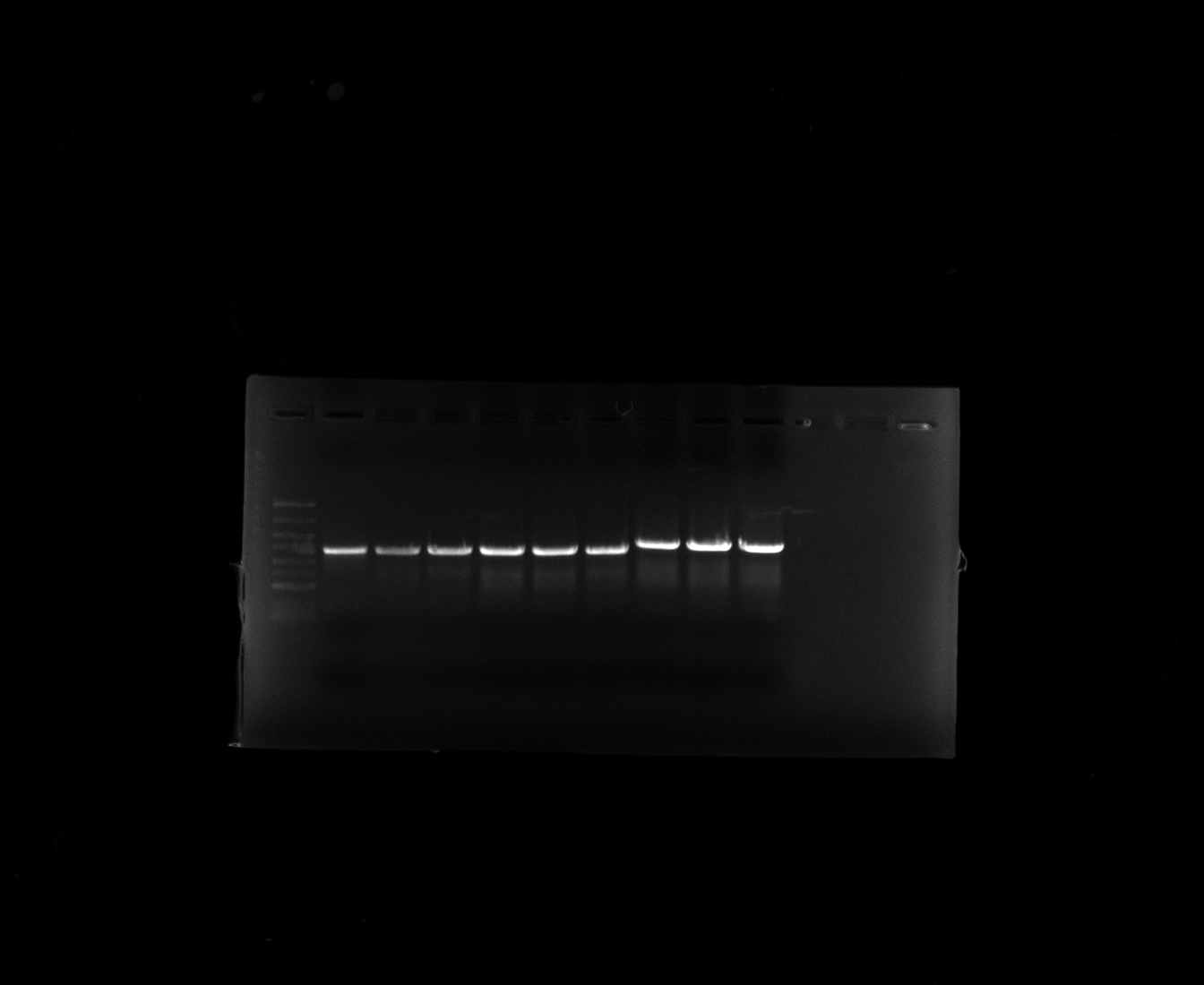

Supplement: Supplementary file 1 [file biology-15-00799-s001.zip › File S1. WB figures/gene of interest.Tif]

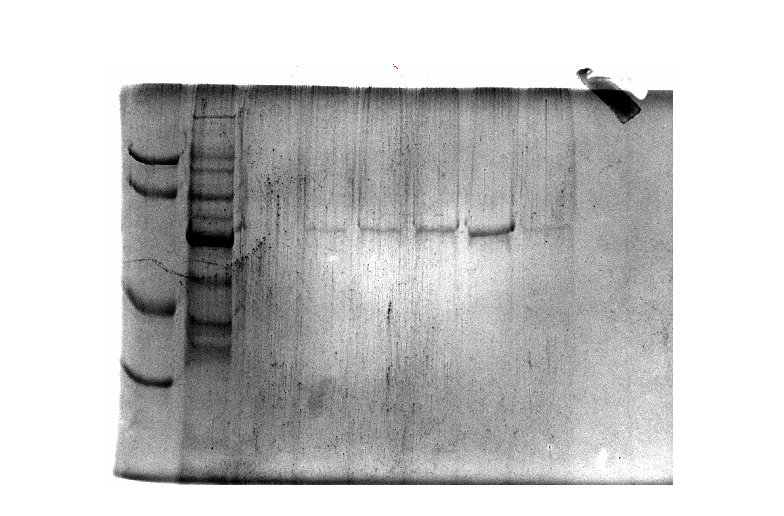

Supplement: Supplementary file 1 [file biology-15-00799-s001.zip › File S1. WB figures/GS enzyme.tif]
